# Giant Protruding Nodular Fasciitis of the Anterior Chest Wall Clinically Mimicking a Soft Tissue Sarcoma

**DOI:** 10.1155/2019/4174985

**Published:** 2019-07-03

**Authors:** Hideyuki Kinoshita, Tsukasa Yonemoto, Hiroto Kamoda, Yoko Hagiwara, Toshinori Tsukanishi, Sumihisa Orita, Kazuhide Inage, Naoya Hirosawa, Seiji Ohtori, Takeshi Ishii

**Affiliations:** ^1^Department of Orthopedic Surgery, Chiba Cancer Center, 666-2 Nitonacho, Chuo-ku, Chiba 260-8717, Japan; ^2^Department of Orthopedic Surgery, Graduate School of Medicine, Chiba University, 1-8-1 Inohana, Chuo-ku, Chiba 260-8670, Japan

## Abstract

Nodular fasciitis (NF) is a benign reactive proliferation of myofibroblasts that predominantly occurs subcutaneously. Commonly, it presents as a rapidly growing swelling in 4-8 weeks. NF mostly occurs in adults aged 20-50 years and usually has a diameter < 3‐4 cm. Giant NF with a diameter > 4 cm is rare. Owing to its rapidly growing nature, a precise clinical diagnosis is difficult; it is frequently misdiagnosed as an aggressive or malignant tumor. Herein, we present the case of a 15-year-old male who presented with a large protruding mass on the anterior chest wall. The tumor appeared clinically malignant as it was protruding and had doubled in size within a few weeks, reaching approximately 8 × 6 cm. Furthermore, the tumor separated and fell off spontaneously due to its large size. As the remaining tumor continued to grow rapidly, surgery was performed. Following wide tumor resection, no recurrence, metastases, or other complications were noted 1 year postsurgery. NF was diagnosed after pathological evaluation, including immunohistochemical analysis, molecular genetic testing, and cytogenetic testing via fluorescence in situ hybridization analysis. Knowledge of the atypical clinical course and a combination of histopathological examinations are necessary to accurately diagnose NF.

## 1. Introduction

Nodular fasciitis (NF) is described as a benign reactive proliferation of myofibroblasts. The exact etiology is still unknown, but a number of hypotheses regard preceding trauma or local irritation as an etiologic factor [1]. NF is fairly common in the soft tissues and most often presents as a small, solitary, and occasionally painful subcutaneous nodule that develops rapidly, often in 4-8 weeks. Contrastingly, NF rarely protrudes from the body surface [2]. Furthermore, NF lesions are usually less than 3-4 cm in diameter. Due to its rapidly growing nature, a precise clinical diagnosis is difficult, and the condition is frequently misdiagnosed as an aggressive or malignant tumor [3]. Recently, *USP6* fluorescence in situ hybridization (FISH) analysis has been considered an important diagnostic tool for NF in conjunction with clinical and cytological findings [4].

Herein, we present a case of giant protruding NF of the anterior chest wall, clinically suspected to be a malignant tumor. Diagnosis and treatment were difficult due to the discrepancy between clinical features, such as the size and shape of the tumor, and the initial pathological results.

## 2. Case Presentation

**Figure 1:**
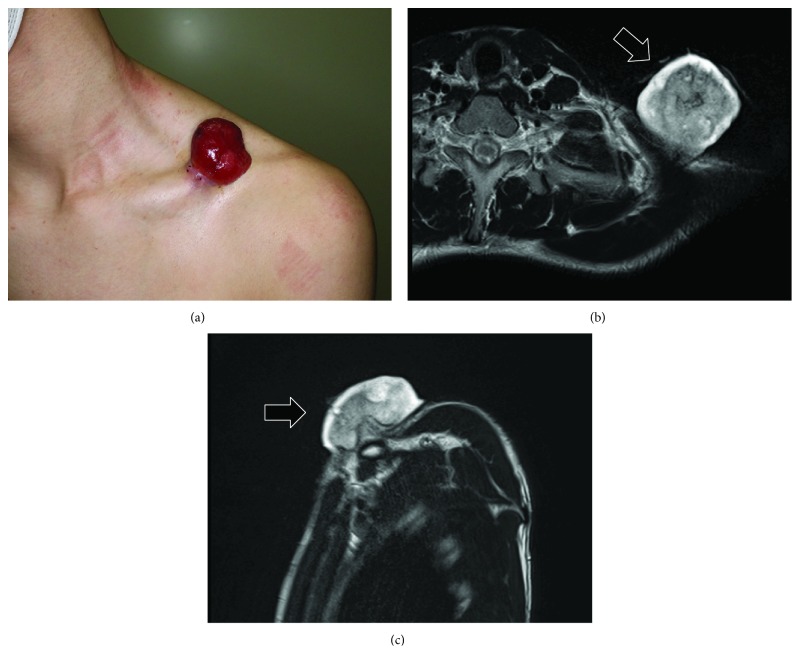
(a) Frontal view of the large protruding tumor, located on the anterior chest wall, measuring 4 × 3 cm at the first visit. (b, c) Axial (b) and sagittal (c) view of the tumor (arrow) on T2-weighted magnetic resonance imaging.

**Figure 2:**
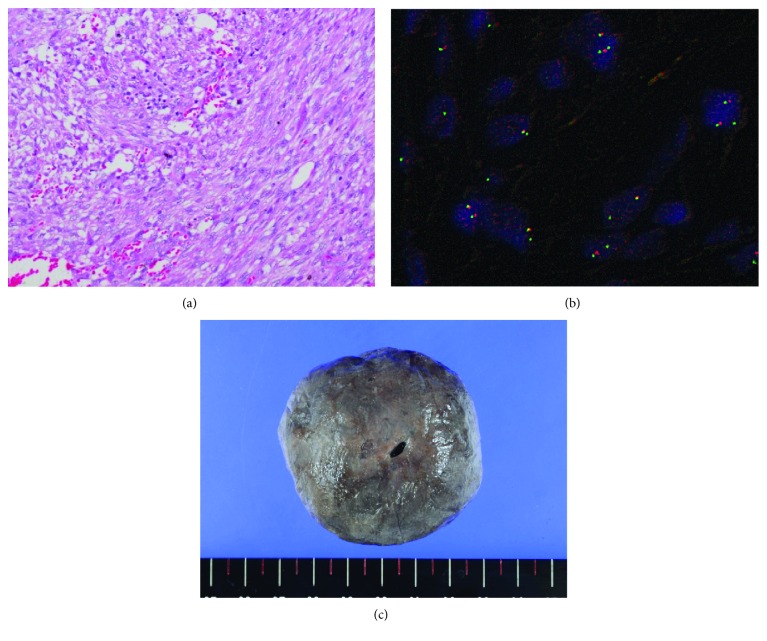
(a) The mass comprising fibromyxoid tissue with focal spindle cell proliferation and inflammation in a loosely myxoedematous matrix with extravasated red blood cells on hematoxylin and eosin (HE) staining. (b) Fluorescence in situ hybridization (FISH) analysis showing *USP6* gene break-apart signals. (c) The tumor with necrosis that spontaneously separated and fell off (measuring 6.5 × 5.3 × 3 cm).

**Figure 3:**
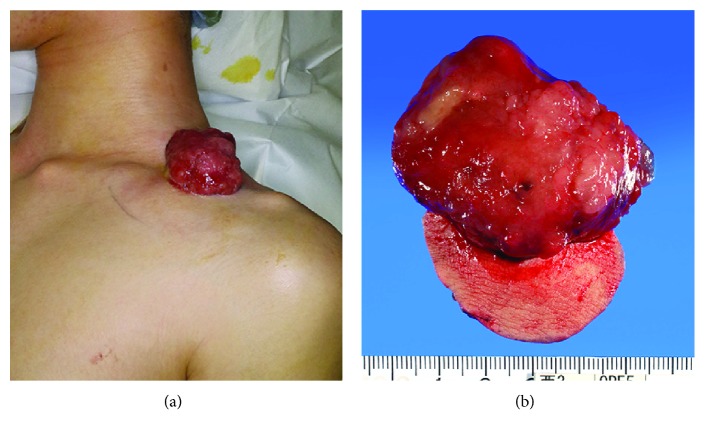
(a) Frontal view of the enlarging tumor with bleeding during the operation. (b) Wide excised specimen of the tumor, measuring approximately 5.5 × 4.5 × 3 cm.

A 15-year-old male patient presented to our orthopedic outpatient department with a rapidly enlarging protruding tumor on the left chest anterior wall, which had gradually increased in size over the preceding year. He had no history of preceding trauma or local irritation. The tumor was asymptomatic but was a physical obstacle due to its protruding nature. On examination, a protruding lesion was noted on the left chest wall, measuring approximately 4 × 3 cm in size (Figure 1(a)). Magnetic resonance imaging revealed a tumor measuring 4 × 3 × 3 cm, protruding subcutaneously superior to the clavicle (Figures 1(b) and 1(c)). Computed tomography revealed that the tumor had not invaded the clavicle. Histopathological examination following hematoxylin and eosin staining of the needle biopsy specimen revealed that the mass comprised fibromyxoid tissue with focal spindle cell proliferation and inflammation in a loosely myxoedematous matrix with extravasated red blood cells (Figure 2(a)). Further diagnostic testing via FISH analysis with a USP6 Dual Color Break Apart Probe was performed. FISH analysis revealed many *USP6* rearrangements by splitting of the red and green signals in both nuclei in this field, strongly suggesting a diagnosis of NF (Figure 2(b)). Although the histopathological results did not match the clinical course, such as extremely rapid growth, he was followed up without treatment. The tumor size rapidly increased to 8 × 6 cm, and the tumor protruded further in a few weeks. One month later, the tumor spontaneously separated and fell off (Figure 2(c)). The separated tumor measured approximately 6.5 × 5.3 × 3 cm and was mostly a necrotic lesion with bleeding. Following this, the remaining tumor on the chest wall continued to grow rapidly (Figure 3(a)).

Although histopathological results confirmed the diagnosis of NF, the clinical findings, which included a protruding lesion that separated and fell off spontaneously, large tumor size, and rapid enlargement, suggested the possibility of malignancy, prompting the need for wide resection. Intraoperatively, we observed that the tumor had not invaded the muscle and bone. The postoperative period was uneventful. The resected tumor measured approximately 5.5 × 4.5 × 3 cm (Figure 3(b)). Histopathological examination of the resected tumor following hematoxylin and eosin staining and FISH analysis with a USP6 Dual Color Break Apart Probe showed similar results as that of histopathological examination of the preoperative needle biopsy specimen. Furthermore, the fusion gene *USP6-MYH9* was confirmed by polymerase chain reaction. These findings were consistent with a diagnosis of NF. After 1 year of follow-up, the patient did not show any recurrence, metastases, or other complications.

## 3. Discussion

NF is a benign reactive soft tissue lesion, frequently found in the subcutaneous tissues and muscle fascia, which shows proliferation of myofibroblasts and fibroblasts, and was first reported by Konwaller et al. in 1955 [5]. Most lesions are solitary and occur in adults 20–50 years of age and have equal incidence among sexes [6]. Reported lesions are usually less than 3–4 cm in diameter and rarely increase in size to more than 4 cm in diameter as occurred in the present case [7]. Bernstein et al. reported that lesions of NF rarely exceed 4 cm and 71% were smaller than 2 cm in their series comprising 134 cases [8]. Lesions may be found anywhere on the body but most commonly on the forearm (27-29%), back or chest wall (15-18%), and upper arm (12%) [3]. Intravascular fasciitis and cranial fasciitis are known subtypes of NF [9, 10]. While it is believed that local injury may play a role in fibroblastic proliferation, one study showed that only 10% of patients described a history of trauma [11]. Clinically, most patients have a history of a rapidly growing mass or nodule that has been present for only 1-2 weeks [12]. Although Majidi et al. reported a protruding NF of the auricle, NF rarely protrudes from the body surface [13]. In the current case, without a history of trauma, after the tumor gradually grew over one year, it grew rapidly into a huge protruding lesion in one month, suggesting the possibility of a malignancy. On a computed tomography scan, NF is seen as a relatively well-defined, soft tissue mass located superficially. On magnetic resonance imaging, NF has been described as inhomogeneous, hypointense to muscle on T1-weighted images, and hyperintense to fat on T2-weighted images, with variable enhancement.

NF lesions are classified into three subtypes according to the anatomical location: subcutaneous, fascial (intermuscular), and intramuscular. The subcutaneous form is most common, followed by the fascial form, with the intramuscular form being the least common [7]. Histologically, NF is usually a well-circumscribed yet nonencapsulated mass composed of fibroblastic and myofibroblastic proliferation arranged in short fascicles and untidy bundles. Typical histologic findings include a myxoid and, in some cases, highly cellular stroma, exhibiting abundant mitotic activity, with the absence of cellular atypia. Myxoid degeneration may also be observed, while other areas are more fibrotic and hyalinized, displaying evidence of adjacent microhemorrhage with granulation tissue [14]. Differential diagnoses are sarcoma with differentiation to myofibroblasts, such as low-grade myofibroblastic sarcoma, undifferentiated pleomorphic sarcoma, and deep fibrous histiocytoma. When it is difficult to diagnose definitely even with hematoxylin and eosin staining and immunohistochemical analysis, molecular genetic or cytogenetic testing for *USP6* genetic rearrangements can be helpful to confirm the diagnosis [15]. In the present case, the lesion was diagnosed as NF with molecular genetic FISH analysis for *USP6* genetic rearrangements. Shin et al. reported that *USP6* FISH is a useful ancillary test in cases where NF is a potential diagnostic consideration [16].

Treatment options include observation and intralesional injection of steroids as spontaneous regression has been reported [1]. Although local marginal excision is by far the treatment modality most commonly opted for, chemotherapy and radiation therapy are not indicated. As recurrence after excision is very rare, in recurrent cases, other malignancies should be considered.

The diagnosis of NF is very challenging, often misdiagnosed as a sarcoma due to its rapid growth, rich cellularity, high mitotic activity, and poorly circumscribed nature. Plaza et al. reported that two-thirds of their cases had been misdiagnosed as sarcoma [17]. Therefore, the knowledge of the atypical clinical course of NF and a combination of these histopathological examinations including FISH analysis with a USP6 Dual Color Break Apart Probe are necessary for an accurate diagnosis.

In conclusion, the present report describes a case of giant NF protruding from the anterior chest wall, which was clinically suspected of being malignant owing to its atypical clinical course and appearance.
